# A Data Quality Control Method for Seafloor Observatories: The Application of Observed Time Series Data in the East China Sea

**DOI:** 10.3390/s18082628

**Published:** 2018-08-10

**Authors:** Yusheng Zhou, Rufu Qin, Huiping Xu, Shazia Sadiq, Yang Yu

**Affiliations:** 1 State Key Laboratory of Marine Geology, Tongji University, Shanghai 200092, China; zhou-yusheng@foxmail.com (Y.Z.); xuhp@idsse.ac.cn (H.X.); yyu@tongji.edu.cn (Y.Y.); 2 School of Information Technology and Electrical Engineering, The University of Queensland, St. Lucia, QLD 4072, Australia; shazia@itee.uq.edu.au; 3 Institute of Deep-Sea Science and Engineering, Chinese Academy of Sciences, Sanya 572000, China

**Keywords:** seafloor observatory, data quality control, ARIMA, outlier detection, data interpolation

## Abstract

With the construction and deployment of seafloor observatories around the world, massive amounts of oceanographic measurement data were gathered and transmitted to data centers. The increase in the amount of observed data not only provides support for marine scientific research but also raises the requirements for data quality control, as scientists must ensure that their research outcomes come from high-quality data. In this paper, we first analyzed and defined data quality problems occurring in the East China Sea Seafloor Observatory System (ECSSOS). We then proposed a method to detect and repair the data quality problems of seafloor observatories. Incorporating data statistics and expert knowledge from domain specialists, the proposed method consists of three parts: a general pretest to preprocess data and provide a router for further processing, data outlier detection methods to label suspect data points, and a data interpolation method to fill up missing and suspect data. The autoregressive integrated moving average (ARIMA) model was improved and applied to seafloor observatory data quality control by using a sliding window and cleaning the input modeling data. Furthermore, a quality control flag system was also proposed and applied to describe data quality control results and processing procedure information. The real observed data in ECSSOS were used to implement and test the proposed method. The results demonstrated that the proposed method performed effectively at detecting and repairing data quality problems for seafloor observatory data.

## 1. Introduction

Seafloor observatories, a universally recognized third observation platform for humans, have become the most remarkable trend in international marine science and technology [1]. Designed with all marine equipment under the sea, cabled seafloor observatories use submarine cables to provide power and transmit information between underwater instruments and shore-based stations. This setup permits the acquisition of long-term, real-time, continuous, high-resolution, and numerous data from in situ instruments [2]. Data collected from seafloor observatories have been providing powerful insights into complex oceanographic processes and are widely used in scientific research such as the geo-, bio-, and hydrosphere interactions and their evolution and variability through time [3]. High-quality research depends on high-quality data which, in turn, depends on robust data quality control practices [2]. Automated methods for rapidly identifying and correcting problematic data are essential [4].

China has been actively pursuing the design and construction of a seafloor observation system (ECSSOS) in the East China Sea [5,6] and will perform research and deployment for national long-term seafloor observation systems in the coming decades. The Xiaoqushan Seafloor Observatory is part of ECSSOS and is the first established seafloor observatory in the East China Sea [7,8]. It has performed continuous measurements and satisfactory operations for more than five years. The Zhujiajian Seafloor Observatory, the second part of ECSSOS, was built near Zhujiajian Island on 19 August 2015. The Zhujiajian Seafloor Observatory used a 50-km submarine electro-optical cable to provide power and transmit information between submarine instruments and the shore station. With the construction of ECSSOS and the accumulation of observed data, research on data quality control for this seafloor observatory is urgent.

Several data quality control methods for seafloor observatories have been proposed or are under study in the wake of seafloor observatory projects performed worldwide. North-East Pacific Undersea Networked Experiments (NEPTUNE) Canada and Victoria Experimental Network Under The Sea (VENUS) are the first multi-node cabled ocean observatories in the world [9,10]. They are operated by Ocean Networks Canada (ONC), along with an increasing number of miniature ocean observatories [11]. Over the years, a comprehensive, process-oriented, quality assurance model and a product-oriented data quality control model have been developed and implemented by ONC. Their data quality control includes both automated (e.g., single-sensor range tests, dual-sensor relational tests, spike detection, and gradient steepness) and manual processes (e.g., regular manual review) to test whether the data meet necessary quality requirements [2]. The Ocean Observatories Initiative (OOI), funded by the U.S. National Science Foundation, works to develop the infrastructure for sustained ocean observations at key coastal and open ocean locations [12]. The OOI applied the system level and human-in-the-loop data quality control methods. At the system level, six automated algorithms, i.e., global range, local range, stuck value, gradient, trend, and spike test, are proposed to be run on datasets. Among those algorithms, the global range, spike test, and stuck value test are currently working as designed by OOI. Daily interactive human-in-the-loop approaches to quality control are performed after automated algorithm tests [13,14]. In Europe, the distributed research infrastructure European Multidisciplinary Seafloor and water-column Observatory European Research Infrastructure Consortium (EMSO ERIC) is implementing fixed-point ocean observatories around the European seas to understand the complex interactions between geo-, bio-, and hydrosphere [15].

Apart from seafloor observatories, a variety of methods have also been proposed for data quality control for marine sensors. The Argo program has deployed more than 3000 floats all over the world ocean [16] and applied two levels of quality control procedures for conductivity, temperature, depth (CTD), and trajectory data: real-time automatic checks and a delayed-model test. A set of automated tests including global range, regional range, pressure increase, spike, gradient, stuck value, density inversion, etc. have been applied to the measurements in the first level [17]. Koziana et al. [18] described the automated data quality assurance system and its algorithm library with the use of measurement range and gradient data checks as part of the U.S. Integrated Ocean Observing System (IOOS), while Bushnell described quality assurance and quality control for real-time oceanographic data in [19]. Its well-established process has resulted in eight manuals [20,21,22,23,24,25,26,27] that provide specific quality control tests for a variety of US IOOS core variables of interest. The National Reference Station (NRS) network, part of Australia’s Integrated Marine Observing System (IMOS), has nine stations strategically positioned to observe a significant proportion of the variability of key oceanographic data processed across all of Australia’s continental shelf. The NRS applied an automated procedure for assessing the quality of temperature, salinity, and chlorophyll-a data by deploying regional range (climatology), spike, and stationarity tests [28]. Good et al. presented global quality control procedures for ocean temperature and salinity profiles for version 4 of the Met Office Hadley Centre “EN” series of data sets in their study [29]. Three new quality control checks were added, and 14 quality control checks were applied to the profile data. Unlike the works described above, thresholds should be defined carefully for each of the quality tests to indicate whether the test has been passed. Rahman et al. [30,31] adopted a supervised classification approach and used a multiple classifier framework for data quality assessments of marine sensor networks, which incorporated balancing to address the unfairness in classification towards minority classes. Timms et al. [32] proposed a novel framework for automated data quality assessments, which uses fuzzy logic to provide a continuous scale of data quality. The continuous quality scale then is used to compute error bars on the data. The highlight of their study was to quantify data uncertainty and provide a more meaningful measure of the data’s fitness for the purpose of a particular application. Inspired by Timms’s approach, Smith et al. [33] proposed a Dynamic Bayesian Network (DBN) framework to produce probabilistic quality assessments and represent the uncertainty of sequentially correlated sensor readings. This work showed that the DBN offered a substantial average improvement in replicating the error bars that were generated by experts compared to that of Timms’s approach.

In this paper, we analyzed and defined the data quality problems of the Xiaoqushan Seafloor Observatory, which is the first seafloor observatory of China. These problems may also be found in other Chinese seafloor observatories. To deal with these problems, a data quality control method was proposed for seafloor observatories. The proposed method consists of three parts: general pretest, data outlier detection, and data interpolation. A quality control flag system was also proposed and applied to describe data quality control results and processing procedure information. 

Unlike those previous works, our data quality control method focuses not only on quality assessments but also on data repair, which we argue is the true meaning of quality control. Long-term and continuous measurements of seafloor observatories provide tremendous scientific application value of observed data, while outliers and data gaps can destroy its continuity and reduce the application value of the data. Therefore, we implemented data repair after data quality assessments. Data repair aims to provide alternative reasonable data values for outliers and missing data points. The ARIMA model was chosen and applied to generate predicted values for outlier detection and data interpolation due to its high forecasting accuracy. This model is suitable for forecasting non-stationary seafloor observatory time series. Previous work [34,35,36] has demonstrated that the ARIMA model is effective at network traffic anomaly detection and prediction. In this paper, we extended its application to seafloor observatory data, and improvements were added to the ARIMA model by using a sliding window and cleaning modeling data. Data statistics and expert knowledge from domain specialists were incorporated into the data quality control method. The proposed method was tested and verified by real observed pH and CTD data. 

## 2. Experimental Seafloor Observatory

The East China Sea is a marginal sea over a broad continental shelf located between the largest continent (Asia) and the largest ocean (Pacific) in the world; the major scientific topic addressed within ECSSOS is sea–land interactions. As the first construction phase (in chronological order) of the ECSSOS, the Xiaoqushan Seafloor Observatory was constructed in April 2009 and accumulated a large amount of observed data. The average water depth of this observatory is 15 m. In this paper, we used the Xiaoqushan station as the experimental seafloor observatory.

In situ measurements of the Xiaoqushan Seafloor Observatory were used to monitor physical and sedimentary processes and to study responses of the ocean environment and ecosystem to extreme weather or climate, such as earthquakes and tsunamis. Some instruments and sensors for physical oceanography were installed in the observatory; including CTD; acoustic Doppler current profiler (ADCP); tide and wave gauge sensors; pH; chlorophyll; dissolved oxygen; colored dissolved organic matter (CDOM); rhodamine; CO_2_ and other marine geochemical devices; and geological and geophysical equipment, such as turbidity sensors (optical backscattering sensor, OBS) and ocean bottom seismometers [8]. These sensors collect data continuously daily, and the main measurement parameters and data collection intervals of some sensors are detailed in Table 1. The observatory collects and transmits over 30 MB data to the data center per day [37]. By examining the observed data stored in the SQL server database, it can be determined that the Xiaoqushan Seafloor Observatory data has three main characteristics. (1) Measurement parameters are varied. There were nine sensors installed, and more than 40 parameters were measured directly or calculated; (2) Most of these parameters are univariate time-series data; (3) The sampling frequency is variable and relatively high; the data acquisition interval ranges from 1 s to several minutes.

**Figure 1:**
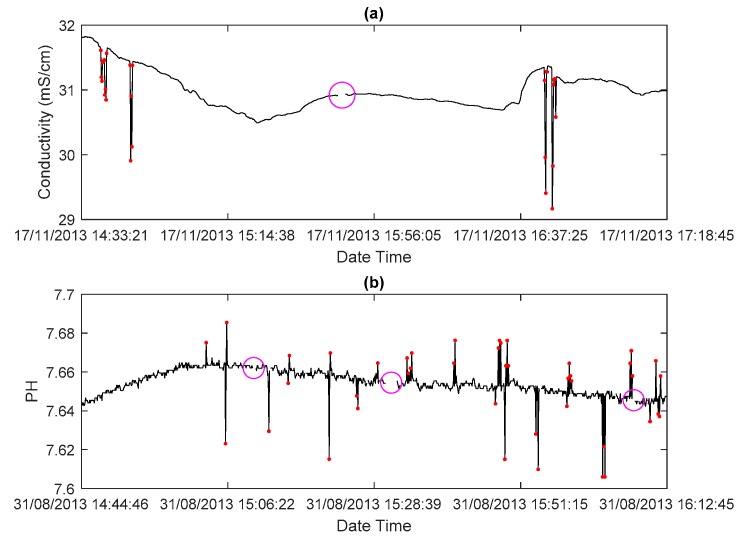
Typical data quality problems in (**a**) conductivity and (**b**) pH observed data series. Red dots denote outliers, and magenta circles denote data gaps.

Sensors deployed in seafloor observatories may degrade in performance and increase measurement deviation through time because of the complex marine environment and long service periods, especially for long-term working chemical sensors. Therefore, sensor calibration before deployment and efficient regular instrument maintenance are very important, which could help to improve the accuracy of observed data in seafloor observatories. However, despite the fact that calibration and regular maintenance have been applied to sensors in the Xiaoqushan Seafloor Observatory, the observed data still have data quality problems based on visual inspection, including outliers, data gaps, and systematic error values (stuck or extreme value). Figure 1 shows data outliers and data gaps in observed conductivity and pH data series. Systematic errors are easy to label, while outliers and gaps should be treated carefully. These are also the focus of this research. Crucial statistic parameters such as maximum value or the hourly average of measurements derived from observed time series with these quality problems can be misestimated significantly. There are many reasons for data quality problems in seafloor observatories. For example, stuck or extreme values can be caused by bio-fouling or sea water corrosion. As the measuring principle of some sensors is based on optics, sensors cannot be covered or corroded. Aging or corroded sensors may produce signal spikes, which often cause data outliers. To our knowledge, sensor failure, data packet transmission failure, and data packet interpretation error are three common causes of data quality problems in seafloor observatories.

## 3. Data Quality Control Method

**Figure 2:**
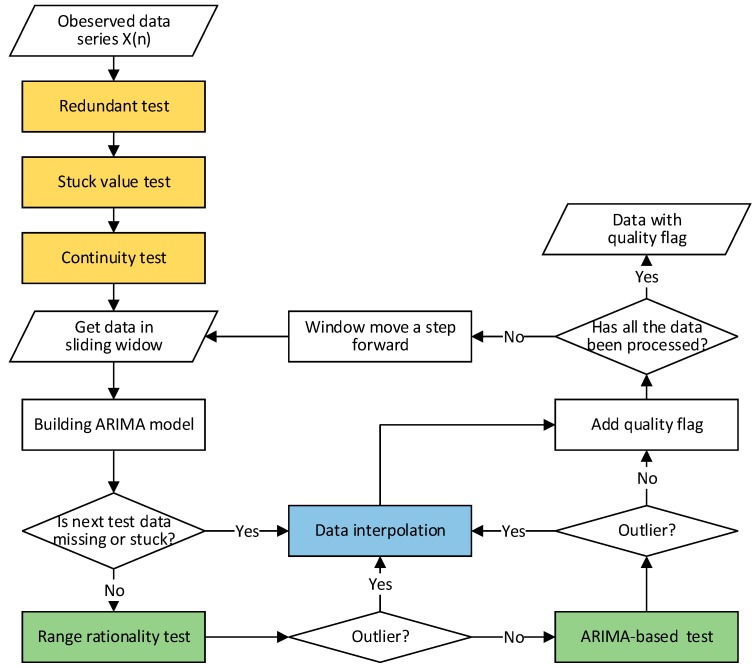
The seafloor observatories data quality control operational framework. Orange denotes the general pretest process, green denotes the outlier detection process, and blue denotes the data interpolation process.

The data quality control method proposed in this paper focuses on time-series observed data, as most of the seafloor observatory data are time-series data. Contraposing the data characteristics and quality problems in ECSSOS, the proposed method consists of three parts: general pretest, data outlier detection, and data interpolation. The seafloor observatory data quality control operational framework is presented in Figure 2.

The entire data quality control method is sliding window-based. The method uses a window of length L to check and repair data quality. Length L is defined according to measurement interval and maximum duration when the measurement assumes no extreme change in the deep sea. The benefits of using a sliding window are obvious. The data within a sliding window before the next test data point are used to model and generate a predicted value for comparison with the observed value. The sliding window improves prediction accuracy due to model refit with the sliding window movement. More accurate predicted data could be conducive to judge whether the data point is normal or abnormal based on the relative error between the predicted and real observed value.

### 3.1. General Pretest

The general pretest tends to perform a quick check for data points. The pretest contains three parts and aims to provide timely data quality information for datasets, as well as to ensure that the data are clean and continuous for further processing. This test detects and deletes redundant data points, labels stuck values, and interpolates missing data with an “NaN” value. The test also decides whether the data gap is suitable for interpolation. Thus, this test could also be regarded as a router to decide the next processing step in the data quality control framework for data points. For example, if a data point was labeled as a stuck or missing value, it will go straight to data interpolation rather than further outlier detection. This test balances the timeliness and completeness of data quality control, as well as saves time for further processing.

#### 3.1.1. Redundant Test

Redundant data in seafloor observatory data are those sample records transmitted to or stored more than once in the database. Many factors result in redundant data in seafloor observatories. The most common reason among them is repeated sensor data package transmission. The number of estimated, repeated, redundant data points in ECSSOS is 5% according to the formal analysis of the observed data. Redundant data points in the database represent a waste of computer storage resources, especially in a seafloor observatory that has an extreme volume of data.

In this paper, we test the differences between neighboring records in acquisition time and measurement value. If these two differences both equal zero, the later record in the time series is regarded as redundant and will be deleted.

#### 3.1.2. Stuck Value Test

The stuck value test checks whether every sampling record is unique and acquired in a normal sensor status. There are sometimes stuck values in the observed time series due to sensor failure or biological pollution. The tolerance for occurrences of stuck values in observed data depends on the measured variable, the sampling interval, and the sensor resolution [28]. The Intergovernmental Oceanographic Commission (IOC) sets the allowable number [38] of consecutive equal values for temperature and salinity:

(1) T=24∗(60/Δt),

in which *T* is the allowable number of consecutive equal values and Δ*t* is the sampling interval in minutes. 

The basis of IOC’s tolerance is the allowable number of consecutive equal values in 24 h. In this paper, we set a predefined tolerance number for repeated occurrences of measurements based on IOC’s principle. A duration of consecutive equal values is defined as *D* for every measurement; the allowable number, *N*, is calculated as follows:

(2) N=D∗(60/Δt),

When tested time series contain constant subsequence and the number of its data points exceeds *N*, these constant data points are labeled as stuck values, and data interpolation should be performed. 

#### 3.1.3. Continuity Test

The continuity test calculates the time gap between two neighboring data points. If the time gap is larger than the sampling interval, it means that there is a gap in the time series and that data interpolation should be performed in this area. The size of the time gap decides the number of interpolated data points. It is inappropriate to do data interpolations when the time gap is too large. According to our experience and data application requirements, if the data gap is larger than 10 min, data interpolation is no longer reliable for raw observed data.

### 3.2. Data Outlier Detection

The common means to recognize outliers is an observation that they deviate far from surrounding observations. This arouses suspicion that it was caused by a different mechanism [39]. Data outliers also follow this definition for seafloor observed data. Data outlier detection for seafloor observatory data aims to flag abnormal data points for data users rather than to reject or delete data. If the observed data value obtains an outlier detection test failure result, we assume that this data value is of bad quality and recommend that it not be used for scientific analysis. The algorithm output provides a simple quality flag, which is stored into related metadata and allows the user to select which data to use.

Two methods are used to detect outliers in this paper. The first method uses expert knowledge from domain specialists, while the second is based on the ARIMA model. The expert knowledge-based method aims to label extreme outliers that exceed a rationality range and will be flagged as bad data. The ARIMA model-based method aims to label those data points that are within the range but are still suspect due to their large deviations from surrounding data points and flag these as suspect data.

#### 3.2.1. Range Rationality Test

A range rationality test evaluates the quality of data points according to whether they fall within a given range. The given range consists of established upper and lower limits based on domain expert knowledge. These limits come from several different aspects. (1) Historical or seasonal expected and valid ranges. This also utilizes site-specific and, possibly, time-varying ranges. Long-term observed data are helpful for setting appropriate limits; (2) Characteristics of observed parameters. Measured data must conform to their physical characteristics (e.g., water temperature must be higher than 0 °C); (3) Characteristics of sensor parameters. Observed data values must not be beyond the sensor measurement range.

To apply this test, the key point is to find a suitable lower and upper limit. Data quality flags could be generated by a simple algorithm that only compares the data point value with the range.

#### 3.2.2. ARIMA-Based Test

Based on the data interpolation method later proposed in this paper, the ARIMA model was also used to detect outliers. The detailed theory and modeling steps for ARIMA are introduced in Section 3.3. Since the ARIMA model could produce a predictive value for every observed data point, outliers can be detected by comparing the predicted value and observed value. The detailed steps are described as follows:

1. Build the ARIMA model with data in the sliding window before the next tested data point.
2. Generate a predicted value for the tested data.
3. Determine whether the tested data is an outlier through the relative error between the predicted and observed value. The tested data point will be flagged as suspect when this calculated relative error exceeds a pre-defined threshold.

### 3.3. Data Interpolation

Data quality assessment is not the ultimate purpose of data quality control, data repair, or data quality improvement. We must do further work to repair suspect and missing data after quality assessment. Data repair does not remove any suspect data but provides alternative data values that are better and more reasonable because of interpolation methods. Scientists can choose interpolated data or just remove suspect data when doing research. In this paper, the window-based ARIMA model is used to perform data interpolation for suspect and missing data.

#### 3.3.1. ARIMA Model

Proposed by Box and Jenkins in the early 1970s [40], the ARIMA model is a well-known and popular method for time series analysis and forecasting applications [41,42]. The ARIMA model originates from the autoregressive (AR) model, moving average (MA) model, and a combination of the AR and MA (ARMA) models [41].

For general time series {x_t_, t = 1, 2, …, n}, the mean is set at E(X_t_) = μ; the AR model of order *p* uses past *p* values in the regression equation, denoted as AR(*p*), which can be expressed as follows:

(3) $$x_{t}=\phi_{0}+\phi_{1}x_{t-1}+\phi_{2}x_{t-2}+\ldots +\;\phi_{p}x_{t-p}+\varepsilon_{t},$$

The MA model of order *q* represents the error of the model as a combination of previous *q* error terms, denoted as MA(*q*), which can be expressed as

(4) $$x_{t}=\mu +\varepsilon_{t}-\theta_{1}\varepsilon_{t-1}-\theta_{2}\epsilon_{t-2}-\ldots -\;\theta_{q}\varepsilon_{t-q}\;,$$

Therefore, the general expression for the ARMA(*p*, *q*) model can be defined as

(5) $$x_{t}=\phi_{0}+\phi_{1}x_{t-1}+\phi_{2}x_{t-2}+\ldots +\;\phi_{p}x_{t-p}+\varepsilon_{t}-\theta_{1}\varepsilon_{t-1}-\theta_{2}\epsilon_{t-2}-\ldots -\;\theta_{q}\varepsilon_{t-q},$$

in which $x_{t}$ is the predicted value at time t, $\phi_{wi}$ are coefficients for each previous observed value $x_{t-i}$, $\theta_{wi}$ are coefficients associated with previous white noises, $\varepsilon_{wt}$ is a white noise series with zero mean, and $\varepsilon_{wt-i}$ are previous noise items.

Generally, the ARMA model is applied to a stationary time series. However, if the series is non-stationary, it can be transformed into a stationary time series by differencing. Therefore, differencing, autoregressive, and moving average components compose an ARIMA model, denoted as ARIMA(*p*, *d*, *q*), which can be expressed as follows:

(6) $$\omega_{t}=\phi_{0}+\phi_{1}\omega_{t-1}+\phi_{2}\omega_{t-2}+\;\ldots +\;\phi_{p}\omega_{t-p}+\varepsilon_{t}-\theta_{1}\varepsilon_{t-1}-\theta_{2}\epsilon_{t-2}-\ldots -\;\theta_{q}\varepsilon_{t-q}\;,$$

in which *ω_t_* = ∇^d^x_t_ and *d* is the degrees of differencing. Note that when *d* = 0, the ARIMA(*p*, *d*, *q*) model degenerates to ARMA(*p*, *q*), then to AR(*p*) and MA(*q*) when *q* = 0 or *p* = 0, respectively.

#### 3.3.2. Advantages of the ARIMA Model

Three major advantages account for the choice of the ARIMA model for data interpolation in seafloor observatory data:

- The ARIMA model originates from the AR model, MA model, and ARMA model [41]. The models could be transformed into each other by appropriate parameter estimation when facing different datasets. This model fully absorbs the advantages of regression analysis and strengthens the good qualities of moving averages [36].
- The ARIMA model can be applied to a non-stationary time series, which is capable of modeling seafloor observatory data, as it is usually non-stationary.
- The computing complexity is affordable, and the accuracy is relatively high when using the ARIMA model for data interpolation and outlier detection in seafloor observatory data.

#### 3.3.3. Improving and Applying the ARIMA Model

In this paper, improvements were added to the traditional ARIMA model before it was applied to seafloor observatory data. First, a fixed size sliding window was used to select historical data for modeling. Since seafloor observatory data are generally stable in a short period but fluctuate over a relatively long term, an appropriate window size can create fast and accurate modeling. The proper window size ensures that the model uses the minimum and effective neighboring previous data and generates a more accurate predicted value, which is good for local outlier detection. Each time the sliding window moves forward a step, the ARIMA model will be updated, i.e., parameters for the ARIMA model will be refitted based on a new dataset within the new window, and a more accurate predicted data value will then be generated. Outliers are replaced by previous predicted reasonable values for the data used to build the ARIMA model. The cleaner input modeling data for the new model ensures the higher accuracy of the predicted value.

Applying a window-based ARIMA model to the seafloor observatory data, we list all steps in the detail as follows. 

Step 1: Obtain L successive data points before the next tested data point.

Step 2: Check whether the selected sequence is stationary using Dickey–Fuller Test. If the sequence is nonstationary, perform the difference until it passes the Dickey–Fuller Test.

Step 3: Establish all possible models based on differencing degree (*D*) and the predefined max order of *p* and *q*. Then, calculate the corresponding AIC (Akaike information criterion). Identify the optimal order of *p* and *q* when the corresponding AIC is the smallest among these models.

Step 4: Generate the predicted value using the selected optimal ARIMA model.

### 3.4. Quality Control Flag

Since the method proposed in this paper does not remove or reject any data points in the seafloor observatory database, additional metadata information about the data quality and processing procedure should be appended to data sample records. In this paper, a quality control flag system was proposed and applied to describe the data quality-related information.

Several regional seafloor observatories around the world have applied flag systems to their data quality. Ocean Network Canada’s flags are based on the Argo quality control flagging system [43] and some ONC-defined flags [2]. The Ocean Observatories Initiative generates quality control flags [14] based on Quality Assurance of Real-Time Oceanographic Data (QARTOD) manuals [44]. Australian National Reference Stations [28] adopted the flag system used by the Intergovernmental Oceanographic Commission (IOC) of United Nations Educational, Scientific, and Cultural Organization (UNESCO) [38,45]. The shared advantage and characteristic of these flag systems is that they are clear and concise. In this paper, we also applied a hybrid flag system based on QARTOD, as well as including some ECSSOS-defined flags (Table 2), which could reveal both data quality and data processing procedure information. The range of flags and descriptions used in this paper are contained in Table 2.

## 4. Application of the Method to Xiaoqushan Observatory

In this research, data collected between August 2013 and September 2014 at the Xiaoqushan Seafloor Observatory were used to test and verify the proposed method. CTD and pH measurements are chosen because of their good continuity and sensor status. Instantaneous measurements are taken every 10 s for CTD and every 8 s for pH. The data are used as follows:

- pH data are used to test and verify the outlier detection method, as outliers occur relatively frequently in pH data. The data interpolation method was applied and verified by CTD measurements.
- All test subset data were selected randomly and distributed evenly throughout the year, and each subset has a certain continuity, which could balance the volume and be representative of seafloor observatory data.
- All test subset data are evaluated manually by domain experts. For outlier detection, manually generated quality flags are used for comparison with flags generated by the proposed method. For data interpolation, manually labeled “correct” data are used to evaluate the data interpolation method, and the predicted value was compared with the actual data point.

### 4.1. Algorithm Design

**Figure 3:**
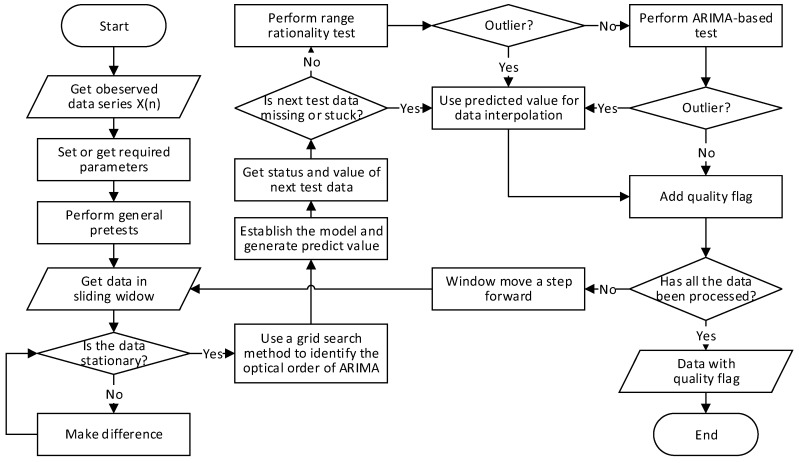
The data quality control algorithm.

Based on the method proposed in Section 3, a complete data quality control algorithm was designed for the Xiaoqushan Seafloor Observatory on RStudio, which uses R version 3.4.4. The whole proposed method algorithm is described in Algorithm 1. Moreover, Figure 3 could help us understand the algorithm.

**Algorithm 1.** The data quality control algorithm.(1)Dat ← getData()(2)errThred ← setErrThred()(3)L ← getWindLen(D, interval)(4)N ← getStuckThred(D, interval)(5)T ← setGapThred()(6)Dat ← delReduntant(Dat)(7)Dat ← labelStuck(Dat, N)(8)Dat ← checkContinuity(Dat, T, interval)(9)while (Dat) do(10)  tbModelDat ← getDatainL(Dat, L)(11)  D ← 0(12)  while (! isStationary(tbModelDat))(13)  tbModelDat ← diffData(tbModelDat)(14)  D ← D + 1(15)  End while(16)  [p, q] ← getBestOrder(tbModelDat, pRange, D, qRange)(17)  Model ← getARIMA(tbModlDat, p, D, q)(18)  predValue ← predict(Model)(19)  [tdatStatus tdatValue] ← getNextTestData(Dat)(20)  If (tdatStatus == missing or tdatStatus == stuck)(21)  Dat ← interpolateData(predValue)(22)  Dat ← setQCFlag()(23)  continue(24)  end if(25)  If (! isInRange(tdatValue, upper, lower))(26)  Dat ← interpolateData(predValue)(27)  Dat ← setQCFlag()(28)  continue(29)  end if(30)  relErr ← errCalculator(tdatValue, predValue)(31)  if (relErr > errThred) Then(32)  Dat ← interpolateData(predValue)(33)  Dat ← setQCFlag()(34)  end if(35)end while

At lines 6–8, three simple functions were used to perform the general pretest. At line 12, the Dickey–Fuller Test was used to check the stationarity of the modeling data and determine the differencing degree for the ARIMA model. At line 16, a grid search method was applied to obtain the best order *p* and *q* for the ARIMA model. The method performed a thorough search for all possible combinations to obtain the minimum AIC, and the corresponding *p* and q were chosen for modeling. At line 20, results of the general pretest were used as a router to decide the next processing step. At line 25, the upper and lower limits based on domain expert knowledge were used to apply the range rationality test. Data interpolation are applied in different situations at lines 21, 26, and 32. Data quality flags are appended according to different data statuses at lines 22, 27, and 33.

### 4.2. Application and Verification

To validate the proposed method, the key point is to evaluate the accuracy of data outlier detection and data interpolation. Applications and their verification of data outlier detection and data interpolation were implemented separately, as data sources and evaluation criteria are different. The general pretest was performed before outlier detection and data interpolation.

#### 4.2.1. Data Outlier Detection

pH data were used to apply and verify outlier detection methods. The test pH dataset was divided evenly into 10 parts according to observed time. A start point then was selected randomly in each part, and the following 1000 successive data points were chosen as one test data subset. The subset time series was included if it contained at least one outlier through visual assessment. Researchers manually labeled outliers in all 10 selected subsets. The number of test data points included altogether is 10,000, in which 441 points were identified manually to be outliers for pH.

To clarify the results of outlier detection methods, true positive (TP), false positive (FP), true negative (TN), and false negative (FN) are defined and explained in Table 3. Positive/negative expresses the detected data point status of abnormal/normal, while true/false means that the detected result is right/wrong when compared with its actual data status.

To evaluate the effectiveness of the outlier detection method, precision (*P*), recall (*R*), and balanced score (*F*1), defined as follows, are used to illustrate the test results.

(7) $$P=\frac{TP}{TP+FP},$$

(8) $$R=\frac{TP}{TP+FN},$$

(9) $$F1=2\times \frac{P\times R}{P+R},$$

Precision is also called the positive predictive value, and recall is also called sensitivity or the true positive rate. *F*1 score is the harmonic mean of precision and sensitivity, which is considered a key criterion. 

#### 4.2.2. Data Interpolation

We applied the method to three CTD measurements, temperature, conductivity, and pressure, to evaluate the proposed data interpolation method. Data interpolation are used in outliers and data gaps. Thus, the method must satisfy two kinds of data interpolation: single-point and successive multipoint interpolation.

For single point interpolation, 3000 data points are chosen randomly in each measurement, and the method is applied to generate 3000 interpolated values. For successive multipoint interpolation, i.e., data gap, 100 time-gap start points are chosen randomly in each measurement, and 30 data values are generated by an interpolation method followed by those start points. Those data points are included only if there are no outliers and missing data appears around them, which ensures accurate input data for interpolation and that the actual data value exists for comparison.

In this paper, we use three criteria to evaluate the proposed data interpolation method: the mean absolute percentage error (MAPE), mean absolute error (MAE), and root mean square error (RMSE). These criteria are calculated as follows:

(10) $$\mathrm{MAPE}=\frac{1}{n}\sum_{i=1}^{n}\left|\frac{x_{i}\left(t\right)-{\hat{x}}_{i}\left(t\right)}{x_{i}\left(t\right)}\right|\times 100\%,$$

(11) $$\mathrm{MAE}=\frac{1}{n}\sum_{i=1}^{n}\left|e\left(i\right)\right|,$$

(12) $$\mathrm{RMSE}=\sqrt{\frac{1}{n}\sum_{i=1}^{n}e^{2}\left(i\right)},$$

in which $e\left(i\right)=x_{i}-{\hat{x}}_{i}$, $x_{i}$ is the actual observed data, and ${\hat{x}}_{i}$ is the interpolated value.

The MAPE is used widely as the basic parameter in such evaluations [46] and is defined as the mean of the absolute percentage differences between the interpolated data value and original observed data. Therefore, the MAPE is considered a key criterion in this paper for evaluating the effectiveness of the interpolation method.

## 5. Results and Discussion

Results were described in this section according to the application of the proposed method for the Xiaoqushan Seafloor Observatory. Firstly, the general pretest was applied to all test data before outlier detection and data interpolation. For stuck value tolerance, we set the duration of consecutive equal values as 5 min, and the corresponding allowable stuck value data points numbers are 38 and 30 for pH and CTD, respectively. The largest time gap allowed in the data interpolation is 10 min. The general pretest results show that the repeated redundant data point rate in the test pH data set is 3.45%, and the number is 5.16% in the test CTD data. There is no stuck value in the test dataset, but the occurrence of data gaps is relatively high in both the pH and CTD data. 

**Figure 4:**
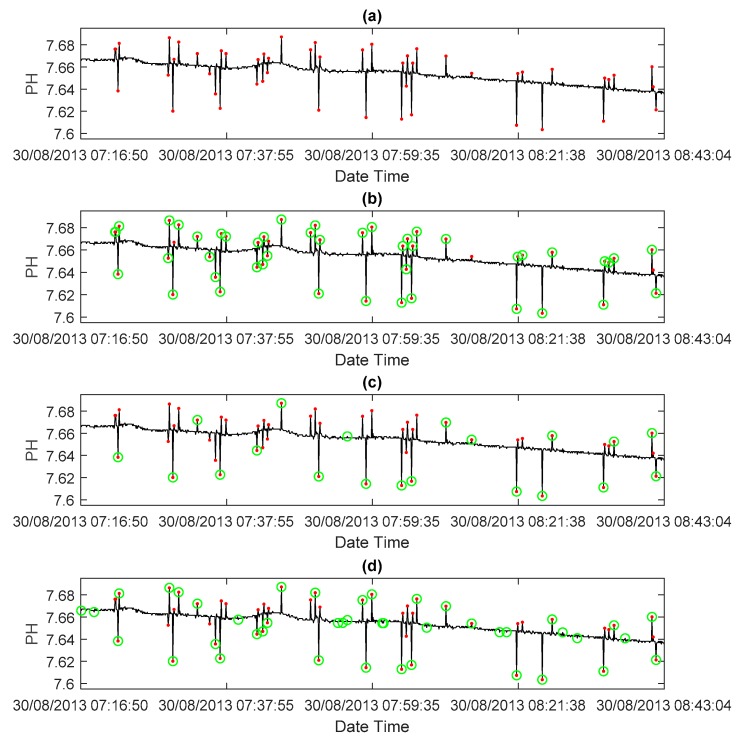
Outlier detection in a subset pH data. (**a**) Original time series, the results of the (**b**) ARIMA method, (**c**) 3sd method, and (**d**) 2sd method. Red dots denote manually labeled outliers, while green circles are outliers detected by the method.

The sliding window size was set to 36 for outlier detection according to the assumption that pH data will not change much in 5 min. We choose 0.065% for the relative error threshold determination for pH data, which depends on different measurement parameters. The standard deviation method, a simple and widely used classical method to detect outliers [47], was also applied to the pH data for comparison. Finally, the results of outlier detection are shown in Table 4. Meanwhile, Figure 4 clearly illustrates the results of outlier detection of different methods in a subset of test data.

It can be observed that the ARIMA method has the best precision, recall, and *F*1 score among all applied methods. When compared with the 3sd method and the ARIMA method, they both have fairly high precision at 0.9628 and 0.9458, respectively. This means that those detected positive status points are mostly are real outliers. Taking recall into consideration, the 3sd method obtained a recall of 0.4354, which is much lower than that of the ARIMA method at 0.9388. The recall result shows that the 3sd method is not sufficiently sensitive, and that this method misses a large number of real abnormal data points. Therefore, the 3sd method had a much lower *F*1 score than that of the ARIMA method. This result of the 3sd method can be associated naturally with the performance of the 2sd method, which may provide a better recall result due to its narrow limit. In fact, the 2sd method performs better when considering the recall, but it also results in lower precision. This means that although more real outliers are detected by the 2sd method, more fake positive statuses occur. The standard deviation method cannot obtain good results for both precision and recall. The 3sd method detects severe outliers but not most of them. The 2sd method detects more outliers at the cost of a high false positive rate. The ARIMA method balances precision and recall and obtains a fairly high *F*1 score in the end, which indicates the effectiveness for outlier detection in pH data by using the ARIMA method.

For data interpolation, the sliding window size was set to 30 according to the assumption that CTD data will not change extremely in 5 min. The accuracy of the MAPE, MAE, and RMSE of the proposed method for each measurement is described in Table 5.

**Figure 5:**
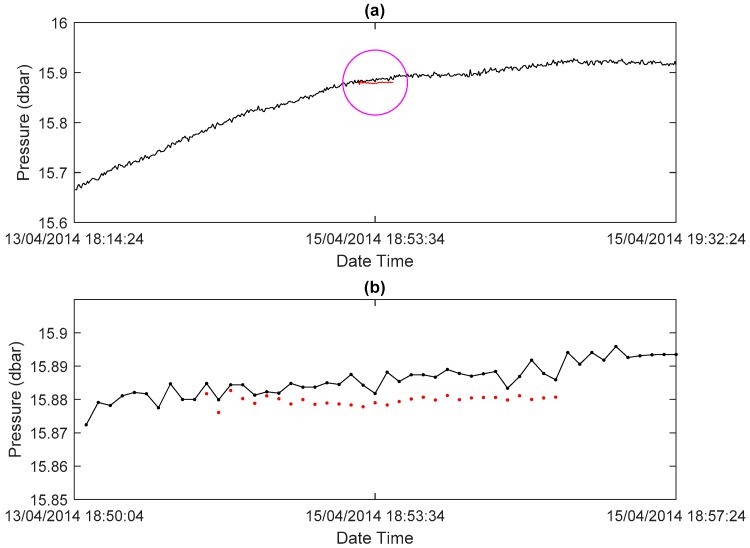
(**a**) An example of data interpolation for successive multipoint in pressure data; (**b**) a partial enlargement of the section of (**a**) in the magenta circle. Red dots denote successive interpolated values.

The MAPE of measurements for single-point interpolation ranges from 0.0015 to 0.0226%. It ranges from 0.0241 to 0.0973% for successive multipoint interpolation. The MAPE is fairly low in these two application situations. This result indicates the high accuracy of the proposed method for both single-point and successive multipoint interpolation. An example of successive multipoint interpolation for pressure is illustrated in Figure 5. Although this is the worst test result (for its highest MAPE), it is clear to see that from the start point, the followed points have similar data values and variation trends between the actual observed and interpolated data. It is also noticed that the deviation is larger when the interpolated data point is farther away from the start point. This observation means that data interpolation should be used carefully in data gaps, especially when the data gap is large. Overall, these results show that the proposed data interpolation method is quite effective for data repair in seafloor observatories data.

## 6. Conclusions

The construction and deployment of seafloor observatories around the world have led to a massive increase in the quantity of oceanographic measurement data. For the data to be fit for research purposes, they must be presented with good quality. 

In this paper, we have designed and implemented a data quality control method for seafloor observatories. The proposed method has been applied successfully for detecting and repairing data quality problems for observed data from the Xiaoqushan Seafloor Observatory. In the case of pH and CTD data, the method detailed in this paper obtained an *F*1 score of 0.9506 in outlier detection and a fairly low MAPE when comparing the actual observed and interpolated data values. These results demonstrated that the proposed method effectively detected and repaired data quality problems for seafloor observatory data. It is also noticed that the proposed method is sliding window-based and backward, which means that it only relies on surrounding data before the tested data point. Therefore, considering its affordable computing complexity and timely response, the proposed method is also capable of being applied in real-time observed data. The method outlined here has been applied to ECSSOS, and it can be regarded as a fundamental framework to address data quality problems in seafloor observatories.

In the future, more improvements such as an adaptive threshold will be added to the ARIMA model for outlier detection, as the relative error threshold used in this study needs to be pre-defined for each measurement. We will also study unsupervised methods to improve the robustness and adaptability of outlier detection in the seafloor observatory data. A systematic seafloor observatory data quality control method and framework, as well as a software system with a graphical user interface (GUI), will be implemented.

## Figures and Tables

**Table 1:** Main measurement parameters and data collection intervals of some sensors.

Sensors	Main Measurement Parameters	Data Collection Intervals (s)
ADCP	depth, current velocity and direction	10
CTD	temperature, salinity, depth	10
CO_2_ Pro	CO_2_	6
Hydrolab DS 5	PH, chlorophyll-a	8
PBR Tide-Wave Recorder	depth	1
Magnetometer	magnetic data	1
OBS-3+	turbidity	10
Ocean Bottom Seismometer	earth motion data	0.01

**Table 2:** ECSSOS data quality control flagging system.

Quality Flag	Description
1	Good data, passed all tests.
2	Data quality control procedures are not performed.
3	Suspect data, failed the ARIMA model-based test.
4	Bad data, failed the range rationality test.
5	Stuck value data.
7	Interpolated single data point by the ARIMA method.
8	Interpolated successive multi data points by the ARIMA method.
9	Missing data.

**Table 3:** Definition of TP, FN, FP, and TN.

Actual Data Status	Tested Results	
	Abnormal	Normal
Abnormal	True Positive (TP)	False Negative (FN)
Normal	False Positive (FP)	True Negative (TN)

**Table 4:** Data outlier detection effectiveness of different methods.

Methods	TP	FN	FP	TN	Precision	Recall	*F*1
ARIMA	414	27	16	9543	0.9628	0.9388	0.9506
3sd method	192	249	11	9548	0.9458	0.4354	0.5963
2sd method	281	160	153	9406	0.6475	0.6372	0.6423

**Table 5:** The data interpolation accuracy of ARIMA for three measurements in two situations.

	Single-Point Interpolation			Successive Multipoint Interpolation		
	Temperature	Conductivity	Pressure	Temperature	Conductivity	Pressure
MAPE	0.0015%	0.0075%	0.0226%	0.0241%	0.0798%	0.0973%
MAE	0.0002	0.0020	0.0032	0.0034	0.0220	0.0140
RMSE	0.0023	0.0060	0.0049	0.0114	0.0687	0.0190
